# Self-rated literacy level does not explain educational differences in health and disease

**DOI:** 10.1186/2049-3258-72-14

**Published:** 2014-05-14

**Authors:** Mirjam P Fransen, Gillian Rowlands, Karlijn EF Leenaars, Marie-Louise Essink-Bot

**Affiliations:** 1 Department of Public Health, Academic Medical Centre, University of Amsterdam, Amsterdam, The Netherlands; 2 Department of Primary Care and Public Health Sciences, King’s College London, London, UK; 3 Institut for Folkesundhed, Aarhus Universitet, Aarhus, Denmark

**Keywords:** Literacy, Health literacy, Literacy measures, Self-rated health, Long term condition, Socio-economic inequalities, Educational inequalities

## Abstract

**Background:**

Although literacy is increasingly considered to play a role in socioeconomic inequalities in health, its contribution to the explanation of educational differences in health has remained unexplored. The aim of this study was to investigate the contribution of self-rated literacy to educational differences in health.

**Methods:**

Data was collected from the Healthy Foundation and Lifestyle Segmentation Dataset (n = 4257). Self-rated literacy was estimated by individuals’ self-reported confidence in reading written English. We used logistic regression analyses to assess the association between educational level and health (long term conditions and self-rated health). Self-rated literacy and other potential explanatory variables were separately added to each model. For each added variable we calculated the percentage change in odds ratio to assess the contribution to the explanation of educational differences in health.

**Results:**

People with lower educational attainment level were more likely to report a long term condition (OR 2.04, CI 1.80-2.32). These educational differences could mostly be explained by age (OR decreased by 27%) and could only minimally be explained by self-rated literacy, as measured by self-rated reading skills (OR decreased by 1%). Literacy could not explain differences in cardiovascular condition or diabetes, and only minimally contributed to mental health problems and depression (OR decreased by 5%). The odds of rating ones own health more negatively was higher for people with a low educational level compared to those with a higher educational level (OR 1.83, CI 1.59-2.010), self-rated literacy decreased the OR by 7%.

**Conclusion:**

Measuring self-rated reading skills does not contribute significantly to the explanation of educational differences in health and disease. Further research should aim for the development of objective generic and specific instruments to measure health literacy skills in the context of health care, disease prevention and health promotion. Such instruments are not only important in the explanation of educational differences in health and disease, but can also be used to identify a group at risk of poorer health through low basic skills, enabling health services and health information to be targeted at those with greater need.

## Background

It has often been shown that those who are worse of in terms of wealth, knowledge and power are also worse off in terms of health. Research on these socioeconomic inequalities in health has progressed over the past few decades and has moved from describing and identifying the problem towards explaining such inequalities and developing interventions to reduce them [1-4].

Literacy may be of theoretical and practical relevance in explaining and decreasing such inequities. Limited literacy is associated with adverse outcomes in health and health care, such as poor knowledge about disease and methods of early disease prevention, high rates of emergency admissions to hospital, and poor self-management of long term conditions [5-12]. Research among elderly in the US even indicated that low literacy was independently associated with a 50% increase of mortality risk [13,14]. In another study among primary care patients with type 2 diabetes, inadequate literacy was independently associated with worse glycemic control and higher rates of retinopathy [10]. The growing awareness that literacy is likely to be a major contributing factor to disparities in health and health care led to research on ‘health literacy (HL)’ in health care, health promotion and clinical research. In a systematic review Sørensen et al. showed that most cited definitions of HL are the ones by the American Medical Association, Institute of Medicine, and the World Health Organisation that focus on individual skills to obtain, process and understand health information and services necessary to make appropriate health decisions [15]. These definitions and measures are closely related to the concept of literacy. With the focus on the role of HL in health promotion, the definition is more expanded into the following categories: (1) basic reading and writing skills that are needed to understand health information (functional HL); (2) advanced cognitive, social and literacy skills that are needed to communicate about health (interactive HL); and (3) advanced cognitive, social and literacy skills that are needed to critically analyze and apply health information in one’s own situation (critical HL) [16]. Some studies consider health numeracy, which refers to one’s understanding and capacity to act on numeric health information, as a fundamental component of HL [17].

**Figure 1:**
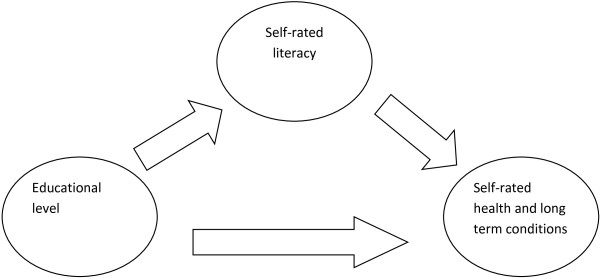
Study objective.

In 2007, the WHO identified literacy as having a central role in socioeconomic inequities in health [18]. Existing conceptual models also propose that (health) literacy is affected by demographic and social factors, such as socioeconomic status (educational level, occupation, employment status) [15]. However, the contribution of both literacy and HL to the explanation of socioeconomic differences in health has remained unexplored. The aim of this study was to assess the role of self-rated literacy in educational inequalities in self-rated health and long term conditions (Figure 1).

## Methods

### Study population and sampling

We used population data from the Healthy Foundations Lifestages Segmentations (HFLS) Survey that was performed in the United Kingdom in 2008 [19]. This survey was organised by the Department of Health (DH) and provided insight into a range of attitudinal, psychological and environmental determinants of health-related behaviour and self-reported health. The aim of the HFLS-survey was to develop a cross-issue segmentation of the population to provide the basis for a strategic approach to health promotion in England. The overall objective was to provide the DH with a coherent view of the nation by segmenting the population into different target audience groups, based on their health motivations and attitudes, personal circumstances and ability to lead healthier lives. In the survey a random sample of people aged 12–75 years in England was recruited to enable detailed analysis among respondents from the most deprived areas, as these areas were more likely to suffer from health disparities. The sample design was such that 60% of the sample was drawn from a nationally representative sampling frame, after stratification by the indices of multiple deprivation, strategic health authority and population density, 40% of the sample was drawn from the 10% most deprived areas in England, following stratification by strategic health authority and population density. A total of 11612 addresses were issued and contacted by phone. After agreement one resident of each household who fell into the eligible age categories was randomly selected to participate in a face-to-face interview. A total of 5380 people were interviewed, representing a net response rate of 55%. For our analyses we used a subsample of 4257 adults aged 25–75 (n = 4257), since education in younger ages may not be fully realised.

### Interview and measures

The questionnaires for the HLFS-survey were assessed in face-to-face interviews by fully trained interviewers. The questionnaire consisted of close and open ended questions on health behaviour and health status, and a self-completion section for potentially sensitive questions. If necessary, professional or informal interpreters were used to provide translation.

For the present analysis we used the following variables:

– Educational level, based on self-reported highest level of attained qualification. Low educational attainment level refers to having no qualifications at all, or having fewer than 5 General Certificate of Secondary Education (GCSE) at grades A-C. The latter is comparable to level 0-2 in the International Standard Classification of Education (ISCED) mapping [20]. Intermediate educational attainment level refers to 5 or more GCDE’s 2 or more A levels, or nursing and teaching qualifications (ISCED level 3-5); High educational attainment level refers to doctorate/masters/bachelor or at least first degree post graduate certificate in education. This is comparable to level 6-8 in the ISCED mapping [20].

– Self-rated literacy, estimated by individuals’ self-reported confidence in reading written English. Respondents were asked ‘How confident do you feel with reading written English?’. Responses were provided on a 4 point scale; not confident at all (1); not confident (2); quite confident (3); very confident (4).

– Ethnic background, assessed by self-identification. Respondents were asked to which ethnic group they considered themselves to belong.

– Long term conditions, assessed by asking the participant whether he or she has a long standing illness, disability or infirmity. If yes, the respondent was asked to indicate the type of illness, disability or infirmity by choosing from a list. Cardiovascular condition was defined as having heart disease/ stroke/ high bloodpressure. Diabetes mellitus was defined as having type I or II diabetes, depression was defined as using medication for depression.

– Self-rated health was measured by the item ‘In general, how would you say your health is?’ Responses were provided on a 5 point scale from ‘very bad’ (1) to ‘very good’ (5).

### Analyses

We used chi-square tests to assess educational differences in health outcomes (long term conditions and self-rated health) and to test the association between educational level and self-rated literacy. We used the method of Baron and Kenny to assess to what extent educational level is related to health, independently of HL [21]. Two sets of logistic regression analyses were performed to assess the contribution of literacy and background variables to the association between educational level and health. The outcome in the first set of regression analyses was ‘having a long term condition or not’. The outcome in the second set of analyses was ‘having a bad self-rated health or not’. For this purpose the variable self-rated health was changed into a binary outcome (‘very bad’ or ‘bad’ versus ‘neutral’, ‘good’, or ‘very good’).

Educational level was added to the model as a binary outcome: ‘low’ versus ‘intermediate/high’. Self-rated literacy was added to the model as an ordinal variable (scale 1–4), age was added to the model as a continuous variable, self-identified ethnicity was added as a binary variable (non UK versus UK). These variables were first separately added to each model and then combined in the final model. Interaction terms were added to the final models to check for effect modification. The change in odds ratio (OR) was used to assess the contribution of each variable to the explanation of educational differences in health.

## Results

### Characteristics of the study population

Table 1 presents the background characteristics of this population (n = 4257). In total 44% of the participants had a low educational attainment level, and 82% of the participants felt very confident in reading English. Most participants (80%) considered themselves British or English.

**Table 1:** Characteristics of the population in the HFLS survey UK in 2008 (n = 4275)

	**N (%)**
Educational attainment level*	
Low	1875 (44)
Intermediate	1266 (30)
High	1016 (24)
Age	
25-34	925 (22)
35-44	952 (22)
45-54	812 (19)
55-64	835 (19)
65-74	751 (18)
Gender (female)	2432 (57)
Self-rated literacy**	
Not confident at all in reading English	77 (2)
Not very confident in reading English	142 (3)
Quite confident in reading English	522 (12)
Very confident in reading English	3529 (82)
Ethnic background	
White British/English	3420 (80)
Other White	218 (5)
South Asian	287 (7)
African/Caribean	226 (5)
Mixed	56 (1)
Other/prefer not to answer	68 (2)

*118 missing.

**5 missings.

### Educational differences in self-rated literacy and health outcomes

Table 2 shows the differences in health outcomes by educational attainment level. Compared to high and intermediate educated participants, those from low educational level more often reported to be in bad health and to have a long term condition. Intermediate educated participants reported to be in worse health and more often had a long term condition compared to high educated participants. All differences were statistically significant (P < 0.05) and accounted for all conditions that are presented in the table.

**Table 2:** Educational differences in health outcomes in HLFS survey UK in 2008 (age 25–74) n (% within educational category)

	**Educational level**		
	**Low (n = 1875)**	**Intermediate (n = 1266)**	**High (n = 1016)**
Self-rated health*			
Good	1281 (68)	970 (77)	853 (84)
Neutral	259 (13)	164 (13)	110 (11)
Bad	333 (18)	129 (10)	52 (5)
Long term condition**			
General LTC	905 (48)	454 (36)	252 (25)
Cardiovascular disease	547 (29)	226 (18)	122 (14)
Stomach, liver, kidney, digestive problems	171 (9)	101 (8)	66 (6)
Diabetes Mellitus (I/II)	173 (9)	65 (5)	36 (3)
Mental health problems/depression/stress	258 (14)	119 (9)	62 (6)
Asthma	246 (13)	134 (11)	84 (8)

*9 missings.

**34 missings.

Table 3 shows the differences in self-rated literacy by educational attainment level. Participants with a lower educational level more often reported not to be confident at all or not to be very confident in reading English than those with a intermediate or higher educational level (9% versus respectively 4% and 1%). Participants with the highest educational level more often reported to be very confident in reading English than those with an intermediate or low level (92% versus respectively 85% and 76%). All reported differences were statistically significant (p = 0.00).

**Table 3:** **Self-rated literacy per educational group in HLFS survey UK in 2008 (age 25–74) *n *** **(% within educational category)**

	**Educational level**		
	**Low (n = 1872)**	**Intermediate (n = 1266)**	**High (n = 1015)**
Self-rated literacy*			
Not confident at all reading English	66 (4)	6 (1)	2 (0)
Not very confident reading English	93 (5)	36 (3)	9 (1)
Quite confident reading English	283 (15)	146 (11)	70 (7)
Very confident reading English	1430 (76)	1078 (85)	934 (92)

*4 missings.

Group differences tested by chi-square (150.6; p = 0.00).

### Association between educational level and long term conditions

Table 4 presents the results of the logistic regression analyses for the following dependent variables: Long term condition in general; cardiovascular disease; diabetes mellitus; and mental health problems/depression. The analyses showed a strong association between educational level and having a long term condition in general (model 1.1; OR 2.04; CI 1.80-2.32). The OR decreased by 1% when literacy was added to the model (model 1.2). When the variable ‘age’ was added to the model (model 1.3), the OR decreased to 1.49 (27%), meaning that these educational differences could mostly be explained by age. When all variables were added to the final model (model 1.6), the variables education, age, ethnic background and literacy remained statistically significant. The final model with interaction terms (not presented in the table) showed that the term age*educational level was not significant, meaning that the effect of age is comparable for the low and higher educated groups. The interaction term self-identified ethnicity*educational level was significant, meaning that the effect of ethnicity on having a long term condition differed per educational group. Ethnicity could therefore be considered as an effect modifier in these models. All other interaction terms were not significant.

**Table 4:** Logistic regression analyses for long term conditions in HLFS survey UK in 2008 (age 25–74) odds ratio (OR)

	**OR (95% CI)**
**Long term condition in general**	
Model 1.1: Educational level	2.04 (1.80-2.32)
Model 1.2: Educational level + literacy	2.02 (1.78-2.29)
Model 1.3: Educational level + age	1.49 (1.30-1.70)
Model 1.4: Educational level + ethnicity	1.95 (1.30-1.68)
Model 1.5: Educational level + gender	2.07 (1.82-2.35)
Model 1.6: Educational level + literacy + age + ethnicity + gender	1.37 (1.19-1.58)
**Cardiovascular condition**	
Model 2.1: Educational level	2.21 (1.90-2.57)
Model 2.2: Educational level + literacy	2.28 (1.95-2.65)
Model 2.3: Educational level + age	1.24 (1.05-1.47)
Model 2.4: Educational level + ethnicity	2.12 (1.82-2.46)
Model 2.5: Educational level + gender	2.22 (1.91-2.57)
Model 2.6: Educational level + literacy + age + ethnicity + gender	1.20 (1.01-1.34)
**Diabetes Mellitus**	
Model 3.1: Educational level	2.10 (1.63-2.68)
Model 3.2: Educational level + literacy	2.09 (1.62-2.69)
Model 3.3: Educational level + age	1.31 (1.01-1.70)
Model 3.4: Educational level + ethnicity	2.10 (1.63-2.70)
Model 3.5: Educational level + gender	2.18 (1.69-2.79)
Model 3.6: Educational level + literacy + age + ethnicity + gender	1.37 (1.04-1.80)
**Mental health/depression**	
Model 4.1: Educational level	1.87 (1.53-2.29)
Model 4.2: Educational level + literacy	1.78 (1.45-2.18)
Model 4.3: Educational level + age	2.05 (1.67-2.53)
Model 4.4: Educational level + ethnicity	1.81 (1.49-2.22)
Model 4.5: Educational level + gender	1.83 (1.49-2.23)
Model 4.6: Educational level + literacy + age + ethnicity + gender	1.79 (1.44-2.23)

The regression models for cardiovascular conditions and diabetes mellitus also showed that educational differences in these outcomes could mostly be explained by age: the OR for cardiovascular disease decreased by 44% (model 2.3), the OR for diabetes by 38% (model 3.3). Self-rated literacy did not decrease the OR for having a cardiovascular condition (model 2.2) or having diabetes (model 3.2), meaning that literacy could not explain educational differences in these chronic conditions. The interaction term age*educational level was not significant in the CVD and DM model. The interaction term self-identified ethnicity*educational level was significant in the CVD model.

The OR for poor mental health or having a depression decreased by 5% when literacy was added to the model (model 4.2), suggesting a minimal, but not statistically significant contribution of literacy to educational differences in poor mental health or depression. The contribution of age, ethnicity and gender was minimal as well, the OR hardly decreased after adding age, ethnicity and gender to the model (model 4.3; 4.4; 4.5). The interaction term self-identified ethnicity*educational level was significant, age*educational level was not significant.

### Association between educational level and self-rated health

Table 5 presents the results of the logistic regression analyses with self-rated health as dependent variable. The OR for rating ones own health more negatively was higher for people with a low educational level compared to those with a higher educational level (model 5.1; OR 1.83, CI 1.59-2.10). The contribution of self-rated literacy (model 5.2), age (model 5.3), ethnicity (model 5.4) or gender (model 5.5) to explaining educational differences in self-rated health was minimal. The ORs decreased by 7%, 8%, 0%, and 0% respectively. The final model with interaction terms (not presented in the table) showed that only the term age*educational level was significant, meaning that the effect of age differs between low and higher educated groups.

**Table 5:** Logistic regression analyses for low self-rated health in HLFS survey UK in 2008 (age 25–74) odds ratio (OR)

	**OR (95% CI)**
Self-rated health	
Model 5.1: Educational level	1.83 (1.59-2.10)
Model 5.2: Education + literacy	1.70 (1.47-1.96)
Model 5.3: Education + age	1.67 (1.44-1.93)
Model 5.4: Education + ethnicity	1.83 (1.59-2.10)
Model 5.5: Education + gender	1.84 (1.60-2.11)
Model 5.6: Education + literacy + age + ethnicity + gender	1.51 (1.30-1.76)

## Discussion

Low educational attainment level was associated with having a long term condition and poor self-rated health. Self-rated literacy could not explain these educational disparities. The association that we found between educational level and health is consistent with the findings in other studies [22-26]. An unexpected finding in our study was the relatively high educational level among people from non-British/English background. This is in contrast to the recent English Skills survey showing that people from ethnic groups other than White British had significantly lower educational levels than White British people [27]. This may indicate either a problem with the purposive sampling for this characteristic or that people with lower education levels were less likely to consent to participation.

The minimal contribution of self-rated literacy in the explanation of educational differences in health raises questions about the potential of literacy measures in research on these socioeconomic inequalities in health. Subjective measures may be less suitable to assess the role of literacy in educational inequalities in health and disease. Subjective measures are prone to the errors associated with any self-report or estimates of behaviours. Patients may be unaware of their lack of ability, or they may overestimate their abilities due to perceived social desirability. Objective, skills-based measures such as the Rapid Estimate of Adult Literacy in Medicine (REALM) [28] or the Newest Vital Sign (NVS) [29] are more promising in this respect. However, a disadvantage of current objective measures is that they only measure the functional component of HL (reading and calculating skills), and not the broader concept of HL that contains interactive and critical skills as well [15,30]. Our findings indicate that measurement of reading skills is insufficient to investigate the role of HL in the explanation of socio-economic inequalities in health.

## Conclusions

Low educational attainment level was strongly associated with reporting a long term condition and poor self-rated health. Differences in general long term condition, cardiovascular condition and diabetes mellitus could mostly, but not completely, be explained by age. Literacy could not explain differences in having a general long term condition, cardiovascular condition or diabetes, and only minimally contributed to mental health problems/depression and self-rated health.

This study is one of the first to investigate the role of literacy in socio-economic health inequalities and makes an important contribution by highlighting the flaws inherent in self-reported literacy skills measures. The same flaws are a weakness of the current study, since we only used self-reported confidence in reading English as an indicator for literacy. Objective measures and measures that assess more than reading skills were not included in this survey. Reading skills are only one component of HL, further research should aim for the development of instruments that objectively measure other components of HL. Such measures have to reach beyond the measurement of basic skills of reading and understanding health information. In the development of such measures, we need conceptual frameworks, such as Sørensen et al’s integrated model of HL [15]. This model describes skills that are related to the process of accessing, understanding, appraising and applying health-related information. These skills also incorporate the qualities of interactive and critical HL, such as the skills to interact effectively with health care providers, or the skills to analyse and compare information to make informed decisions in e.g. screening. In order to capture all relevant aspects of HL in specific contexts in health care, disease prevention or health promotion, it is important that a HL measure is tailored to the skills that individuals need in that specific context in. Individuals that for example are diagnosed with cancer need skills to read and understand information about the diagnosis, skills to seek and extract information about treatment options, skills to express emotions and expectations in interaction with their health care provider, and skills to critically consider pros and cons of treatment options.

Objective skills-based HL measures are not only important in explaining educational differences in health and disease, but can also be used to assess the prevalence of low HL and identify groups at risk of poorer health through low basic skills. This is essential for the development and evaluation of tailored interventions that should be aimed at increasing population and patient empowerment and competencies of health care professionals and health care systems.

## Competing interests

The authors declare that they have no competing interests.

## Authors’ contributions

MF designed and performed the analyses, and wrote the paper. GR performed acquisition for the data, contributed to the design and the analyses. ML contributed to and supervised the design and analyses. KL revised the paper critically. GR, KL and ML been involved in drafting and revising the manuscript and have given final approval of the version to be published. All authors read and approved the final manuscript.
